# Five Years of Designing Wireless Sensor Networks in the Doñana Biological Reserve (Spain): An Applications Approach

**DOI:** 10.3390/s130912044

**Published:** 2013-09-10

**Authors:** Diego F. Larios, Julio Barbancho, José L. Sevillano, Gustavo Rodríguez, Francisco J. Molina, Virginia G. Gasull, Javier M. Mora-Merchan, Carlos León

**Affiliations:** 1 Departamento de Tecnología Electrónica, Universidad de Sevilla, Seville 41011, Spain; E-Mails: jbarbancho@us.es (J.B.); gusrodri@us.es (G.R.); fjmolina@us.es (F.J.M.); vgasull@us.es (V.G.G.); jmmora@us.es (J.M.M.); cleon@us.es (C.L.); 2 Departamento de Arquitectura de Computadores, Universidad de Sevilla, Seville 41012, Spain; E-Mail: sevi@atc.us.es

**Keywords:** wireless sensor network, habitat monitoring, neural networks, computational intelligence

## Abstract

Wireless Sensor Networks (WSNs) are a technology that is becoming very popular for many applications, and environmental monitoring is one of its most important application areas. This technology solves the lack of flexibility of wired sensor installations and, at the same time, reduces the deployment costs. To demonstrate the advantages of WSN technology, for the last five years we have been deploying some prototypes in the Doñana Biological Reserve, which is an important protected area in Southern Spain. These prototypes not only evaluate the technology, but also solve some of the monitoring problems that have been raised by biologists working in Doñana. This paper presents a review of the work that has been developed during these five years. Here, we demonstrate the enormous potential of using machine learning in wireless sensor networks for environmental and animal monitoring because this approach increases the amount of useful information and reduces the effort that is required by biologists in an environmental monitoring task.

## 1. Introduction

A wireless sensor network (WSN) is composed of many small devices that are deployed in a physical environment. Each device, called a node, has special capabilities, such as communications with its neighbors, sensing and data storage and processing [1]. The nodes form mesh networks of devices that can collaborate among themselves, which allows for the implementation of distributed solutions to solve complex problems.

Although sensor networks have many applications [2], environmental monitoring is an area in which the potential impact is extremely large, as described in [3]. These devices permit monitoring an area at a low cost and with little need for human presence, which is very important in these types of applications [4]. These networks are normally used in environmental monitoring to collect information via sensors that are incorporated into each node, and they pool this information in a special device called a Base Station [5]. The Base Station usually sends the information to a main server, where the collected data can be accessible through an infrastructure network, such as the Internet. The technical requirements of a WSN applied to environmental monitoring include:

(1) Autonomy: Batteries must be able to power the nodes during the whole network lifetime. Because the radio transceiver accounts for most of the power consumption in a node, the network must reduce the data traffic as much as possible as well as reducing the number of hops that are required to send a message.
(2) Robustness: In this type of application, human maintenance is usually difficult because of the challenging terrain. Therefore, it is important to design robust software and hardware that can be adapted to any incident.
(3) Flexibility: The network must be able to add, move or remove nodes to meet the application requirements. The network must automatically detect the changes, organizing the communications as a consequence.

Although WSNs solve some common problems in wired environmental monitoring installations, in practical installations, these devices have some challenges that must be overcome. These challenges come from the fact that a WSN is typically formed by very small devices that have several restrictions: low power consumption, low weight (especially for mobile devices), low cost and low processing capabilities.

For many years now, the authors of this paper have collaborated with the Doñana Biological Station. During these years, they have been designing and developing WSNs to be used in this park. In this paper, we describe all of the networks that are used to solve real monitoring problems of the biologists who work in Doñana National park.

The remainder of this paper is organized as follows: Section 2 summarizes the environment in which the networks are deployed. Section 3 describes the networks chronologically. The future networks that are being developed are described in Section 4. Finally, Section 5 presents the concluding remarks.

## 2. Description of the Environment to Monitor

**Figure 1.**
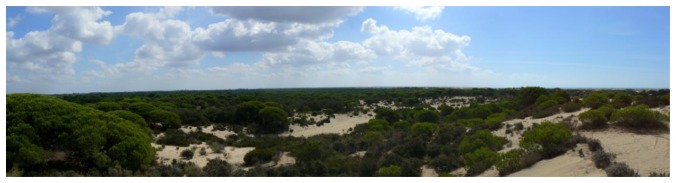
Natural Park of Doñana.

All of the proposed WSNs have been designed to be applied in the Doñana Biological Reserve (DBR). The DBR is part of Doñana Natural Park, which is a wildlife refuge in southwestern Spain. It is located in Andalusia, among the provinces of Huelva, Sevilla and Cadiz, and covers 543 km^2^, of which 135 km^2^ are a protected reserve. The park is an area of marshes, shallow streams, Mediterranean scrub and sand dunes (Figure 1), which are managed by the Doñana Biological Station (DBS), a Research Institute of the Spanish Council for Scientific Research (CSIC). The main goals of the DBS are conservation and improving the quality of research in Doñana, which was declared a World Heritage Site by UNESCO in 1994. This region is considered to be one of the most important natural protected landscapes in the world, and the DBR has been included among the great scientific infrastructures of the European Union. In addition, in April 2006, the Spanish Interministerial Commission of Science and Technology (CICYT) from the Spanish Science and Education Department recognized the Doñana Scientific Reserve as a Singular Scientific and Technological Infrastructure (ICTS).

Because of the relatively minimal human presence in the environment and the high degree of species richness in the area, Doñana is an ideal location for studying wild plants and animals. Many such studies have been conducted over the last 50 years, mostly using “traditional” methods that are based on the direct observation of natural subjects. Only in the past decade has the DBS started to establish facilities that have technologies that allow for the automatic gathering of information without direct human interference.

### 2.1. Description of the Research Facilities in Doñana

Early sensor deployments in Doñana integrated different types of sensors to gather information about underground water levels and vegetation stress, among other subjects. However, these initial installations were run in an *ad-hoc* manner, without integration or communication between the different networks. Moreover, the facilities were based on wired networks.

**Figure 2.**
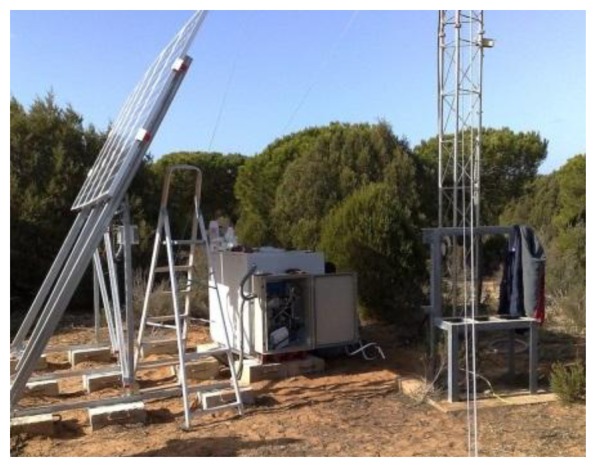
National Park of Doñana.

To allow for the integration of sensors and to facilitate the retrieval of information, some remote communication towers have been currently deployed in the Doñana Biological Reserve (Figure 2). Thus far, there are six remote communication towers that are spread all over the park. These communication towers use a WiMAX connection to allow communication with a central server.

Each communication tower provides a local wired network, Wi-Fi, and a wired datalogger, which allow it to deploy sensors in the surroundings of these towers. Moreover, these towers provide power energy to wired sensors by solar panels.

The Wi-Fi is mainly used for communication with remote motorized cameras. In the current typical installations, most of the remaining sensors are connected to the datalogger by wires. However, the use of wired communication sensors has drawbacks, such as:

- If aerial cables are used, then they are exposed to animals that continuously break the lines.
- The use of underground wires increases considerably the cost of installation and could affect the vegetation of the zone.
- The maximum distance between the sensors and the communication tower is limited by the wired technology that is used (4–20 mA sensors). For this reason, there are some areas of the natural park that do not have coverage for monitoring.

Furthermore, wired installations have an important drawback in their lack of flexibility: after the installation is performed, it is difficult and expensive to add new sensors or to modify their locations, especially in harsh environments such as Doñana. There are important areas of the park that have very difficult access to vehicles, which complicates the transportation of the hardware that is required to modify the wired installations.

## 3. Current WSNs Designed for Doñana

The use of WSNs avoids the aforementioned limitations of their wired sensor installations. WSN devices are easy to deploy and easy to reconfigure. They allow adding new devices easily or changing the location of the existing devices. Moreover, because they are usually small devices, their deployment does not require complex material transportation or complex infrastructure. Wireless technology has less reliability than wired technology, but currently, the modulation mechanism used in WSNs can maintain an acceptable reliability. As will be discussed shortly, in our installations, we obtained a reliability of approximately 80%.

In the past, the use of WSN technology has been limited because of the high cost and reduced coverage of these devices. However, currently, the price of these devices is low enough to allow deployment of extremely large networks. Moreover, we have recently tested some WSN radio transceivers with a coverage area of several kilometers. These radio transceivers work in the RF band of 868 MHz and use a radio signal power amplifier [6]. To demonstrate these advantages, we collaborated with the DBS in 2008, when we proposed the deployment of a WSN prototype in the National Park of Doñana. As a result of this collaboration, some different networks have been designed. These networks will be described in the following sections.

### 3.1. ARTICA 1 Network

**Figure 3.**
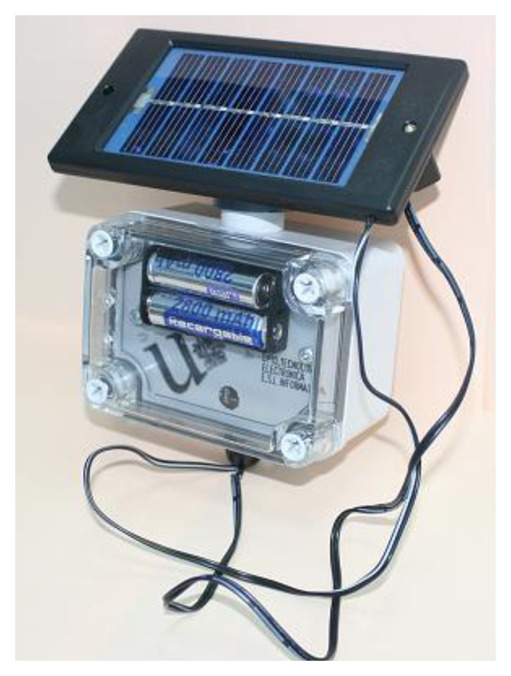
Node of the ARTICA 1 network.

ARTICA 1 was our initial prototype. It was designed in 2008, and it was based on a TelosB platform. Figure 3 shows a node of the ARTICA 1 network. These nodes are powered by small solar panels and are designed to obtain the following measurements:

- Temperature semiconductor sensor in the −40–80 °C range, calibrated to obtain an accuracy of 0.1 °C in the 0–70 °C temperature range.
- Relative humidity, with a semiconductor sensor that works in the 0–100% RH range.
- Solar radiation in visible light (in the [320 nm to 1,100 nm] wavelength range) and NIR (Near-InfraRed in the [320–730 nm] wavelength range) using photodiodes.

These nodes were designed as a general device system that allows distributed and collaborative data processing over the network, using IEEE 802.15.4 2.4 GHz radio transceivers.

**Figure 4.**
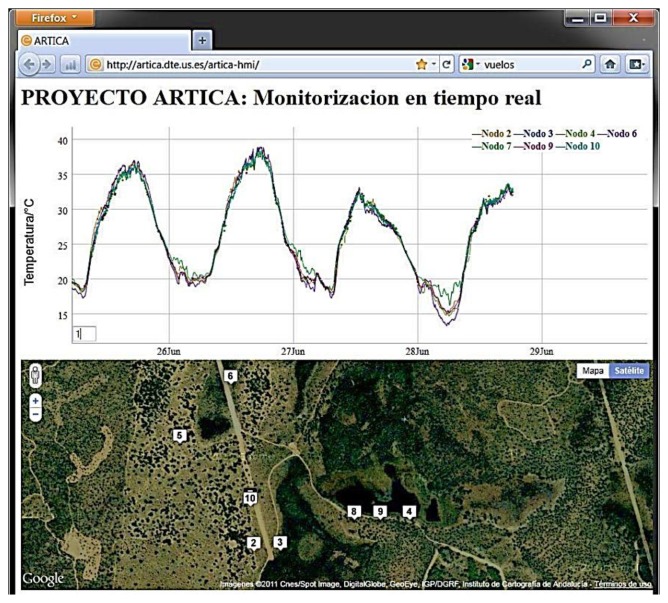
Access GUI to ARTICAs networks.

The initial ARTICA 1 network was composed of four nodes that were spread over an area with a distance of up to 200 m between them. These nodes were designed to read the environmental monitoring information from the sensors every minute, sending the information to them. The maximum distance between nodes is 200 from the central server after its capture. These nodes are being designed to work up to a week in complete darkness. Figure 4 shows a graphical GUI of the environmental information obtained from this network, through the central server.

To allow for reduced power consumption, it is necessary to use adapted communication protocols. Currently, efficient routing protocols with low power consumption continue being a main issue for WSNs; for example, see the recent review in [7]. Several routing solutions have been proposed in the literature [8], which are mainly focused on energy consumption [9,10], security issues [11,12] or fault-tolerant capacity [13].

Currently, the ARTICA 1 network and the subsequent prototypes use a routing algorithm that is based on SOM (Self-Organizing Maps) [14], adding a synchronization mechanism that allows the hibernation of nodes, including the radio transceiver, most or a portion of the time. These networks are working with a sampling time of one measurement from each sensor per minute.

### 3.2. ARTICA 2 Network

**Figure 5.**
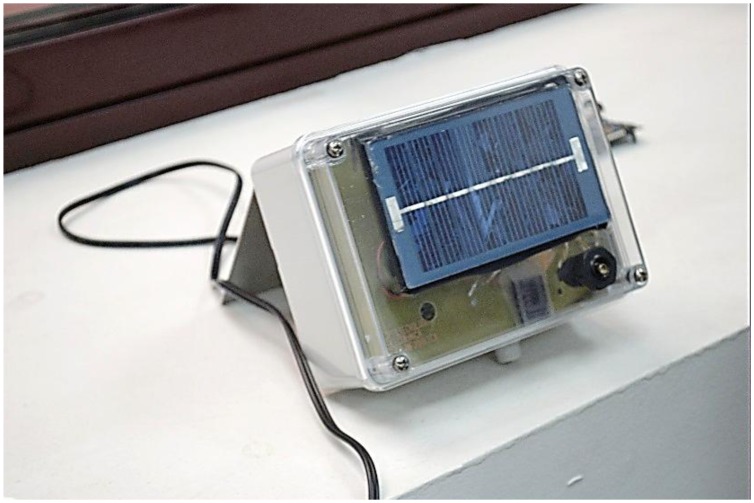
Node of the ARTICA 2 network.

The second prototype, ARTICA 2, was designed in 2009. ARTICA 2 nodes are an evolution of the nodes deployed in the ARTICA 1 network, including an improved power system and an improved sensor interface but using the same processor and radio transceiver technology. Figure 5 shows a node of this network.

The ARTICA 2 nodes integrate the same measurements and the same sampling rate as the ARTICA 1. However, the ARTICA 1 network does not allow adding additional sensors, but ARTICA 2 nodes were redesigned to easily allow the integration of additional sensors by using an external sensor interface. Using this interface, some of the ARTICA 2 nodes have external moisture sensors or external infrared radiometers.

The ARTICA 2 network is composed by six nodes that use the same communication protocol as is designed for the ARTICA 1 network. The ARTICA 1 and ARTICA 2 networks have been used to demonstrate the reliability of the communications in these scenarios. Several tests were developed outdoors, and these tests measure the percentage of packet losses. We obtained a reliability of 80%. This finding means that fewer than 20% of the transmitted packets were lost. This percentage loss is a good result, especially considering the interference that is produced by the vegetation, which blocks transmissions in the 2.4 GHz range.

### 3.3. ICARO 1 Network

**Figure 6.**
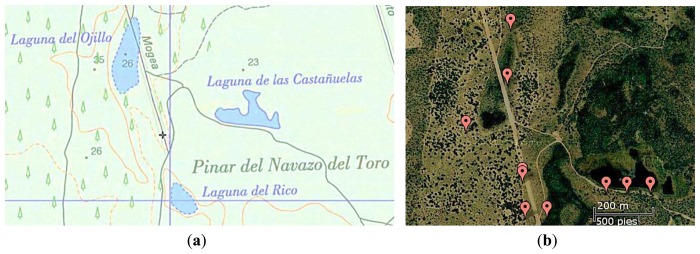
Localization of the ICARO 1 network. (**a**) satellite image, (**b**) topographical map.

Based on our previous experience in WSNs, in 2010, we deployed a WSN to solve a specific problem that the biologist had in Doñana (Figure 6): monitoring the flood level in marsh areas. This network was called ICARO 1. The ICARO 1 network is composed of ten wireless sensors that are deployed between the zone of “El Ojillo” and “El Zacallon” [15]. This network works on the 2.4 GHz ISM band using the IEEE 802.15.4 Protocol. Figure 6 shows a map with the current deployment of the system. The maximum distance between the nodes is 200 m.

**Figure 7.**
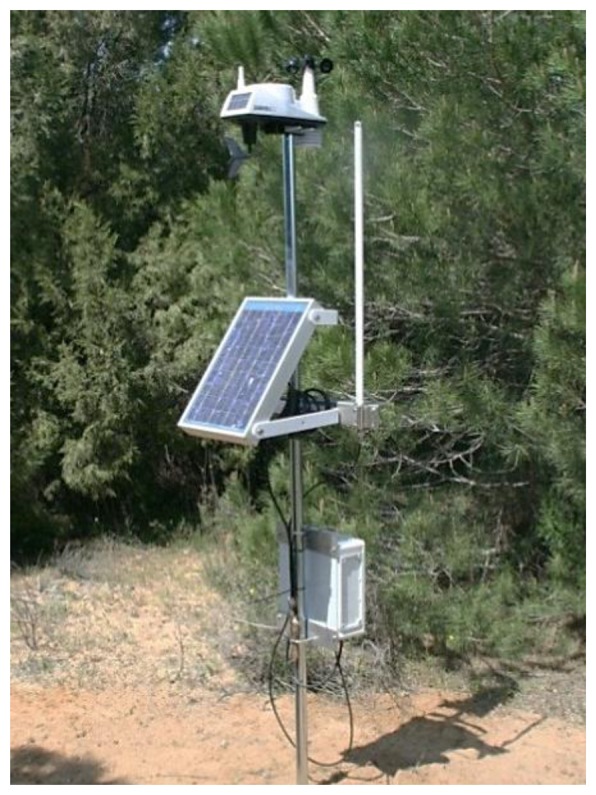
Node of the ICARO 1 network.

The ICARO 1 nodes (Figure 7) are powered by small solar panels, and they are designed to allow the execution of distributed and collaborative algorithms. Data fusion and data aggregation are used to reduce bandwidth usage and power consumption. The nodes of the ICARO 1 network integrate the following sensors:

- Temperature, which is calibrated to obtain an accuracy of 0.1 °C in the −40–65 °C temperature range.
- Relative humidity, which is in the 0–100% RH range.
- Solar radiation (in the visible light range of 320–1,100 nm and the NIR 320–730 nm range, using photodiodes).
- Rainfall level, using a tipping spoon with a resolution of 0.2 mm.
- Wind direction, with one degree of accuracy.
- Wind speed, with an anemometer that works in the 3–240 km/h range.

In this network, the flood level estimation is obtained by using a novel computational intelligence algorithm [15], which is based on the use of SOM maps [14]. This algorithm is designed to optimize the energy consumption and to obtain the flow level information using environmental variables, such as the rain level or average temperature. These variables are acquired every 5 min, but a message is sent only if a meaningful flood level variation is detected. The training of the algorithm is obtained from historical environmental information obtained from the DBS and is evaluated via simulations.

**Figure 8.**
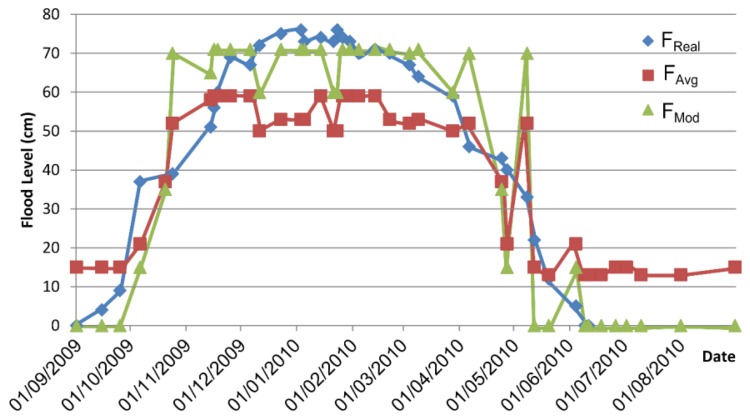
Flood level estimation of the ICARO project.

After its deployment in 2010, the experimental results obtained under various scenarios have demonstrated good performance of the system and have validated the results that were obtained by simulation. This arrangement is depicted in Figure 8, where F_Real_ represents the real flood level, F_Avg_ represents the flood average estimation and F_Mod_ represents the flood mode estimation.

#### 3.3.1. Animal Localization Project

In addition to its use for flood level monitoring, the ICARO 1 network has also been used for other projects. For example, in 2011, it was used to deploy a localization prototype. In this prototype, the ICARO 1 infrastructure of the nodes was used for anchor nodes, which were used together with a newly developed low weight and low cost mobile device that had high autonomy, and it was used as a non-anchor node for tracking animals in the park.

To obtain the localization of non-anchor devices, we proposed the use of an algorithm called *Localization based on an Intelligent System* (LIS) [16]. LIS is a range-free localization technique. This algorithm is useful in applications that have high power consumption limitations. In applications such as wildlife localization in a natural environment, where the power availability and the weight are substantial restrictions, the use of energy-hungry devices that add extra weight, such as GPS's or hardware (for example, a mobile directional antenna) is not a good solution.

**Figure 9.**
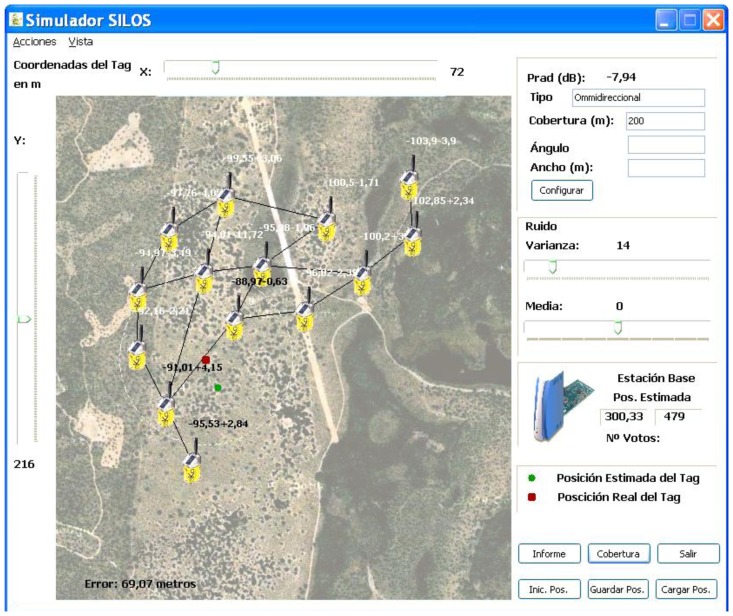
LIS Graphics Environment.

For these reasons, for localization purposes, it would be better to use implicit characteristics in communications, such as connectivity, number of hops or RSSI (Received Signal Strength Indicator). The measurements that are related to these parameters are currently integrated in most radio devices. These measurement techniques are based on the beacons' transmissions between the devices. The LIS algorithm is designed by taking these characteristics into account. Figure 9 shows the graphics environment of the LIS algorithm, which is designed for the ICARO 1 network.

LIS includes tracking with a distributed localization method that uses small mobile nodes (called tags), which have no additional hardware requirements, into a network of anchor nodes. LIS implements a hibernation protocol for the non-anchor nodes, which can save a large amount of energy on these devices.

**Figure 10.**
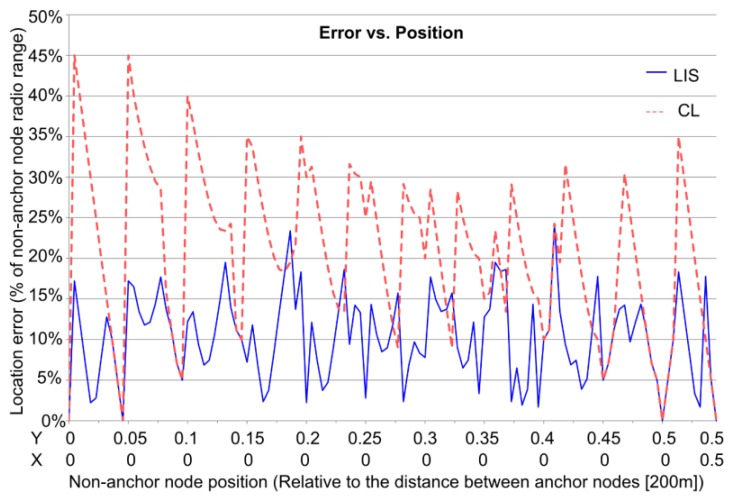
LIS localization errors.

LIS obtains the position with a combination of two algorithms; one algorithm is based on a local node that uses a fuzzy system to obtain a partial solution, and the other algorithm is based on a centralized method, which is executed in a base station or a cluster-head that merges all of the partial solutions. Figure 10 shows the localization error of LIS compared with the Centroid Localization (CL) algorithm [17].

According to our results, this localization technique has been demonstrated to be useful in applications in which external devices such as GPS are not useful because of their high level of power consumption.

#### 3.3.2. Forest Fire Detection Project

Another application in which we used the ICARO 1 network was in the SIPIRSI project [18]. This project is focused on obtaining two types of information: the risk of a fire and, in the case of an active fire, the direction of the flames and their velocity. This information is useful to fire rangers to obtain escape paths and to determine strategies for extinguishing a fire.

**Figure 11.**
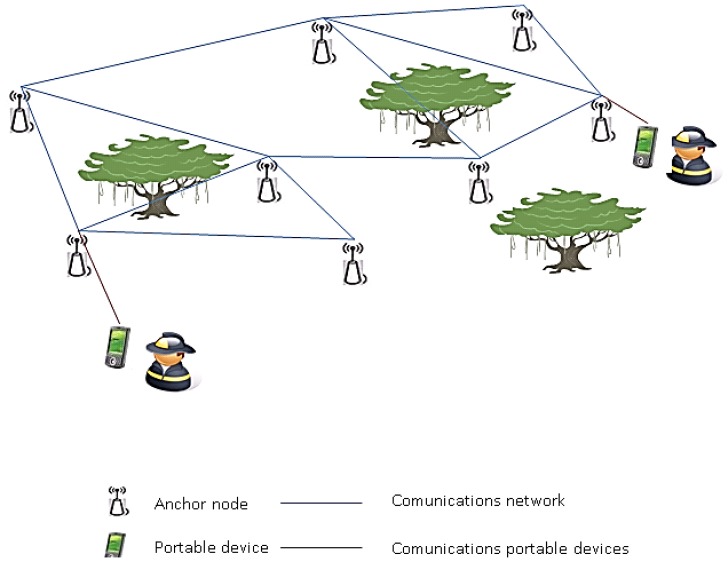
SIPIRSI architecture.

To obtain this information, SIPIRSI uses environmental information such as temperature or wind direction, which is obtained from the sensors of an ICARO 1 network. The structure of SIPIRSI is depicted in Figure 11.

The SIPIRSI algorithm is based on a combination of two methods: one distributed and one centralized, which are both based on fuzzy logic processing [19]. The distributed algorithm is used to obtain a prediction of the risk of fire, while the centralized algorithm is used to estimate the propagation direction, in the case of an active fire.

**Figure 12.**
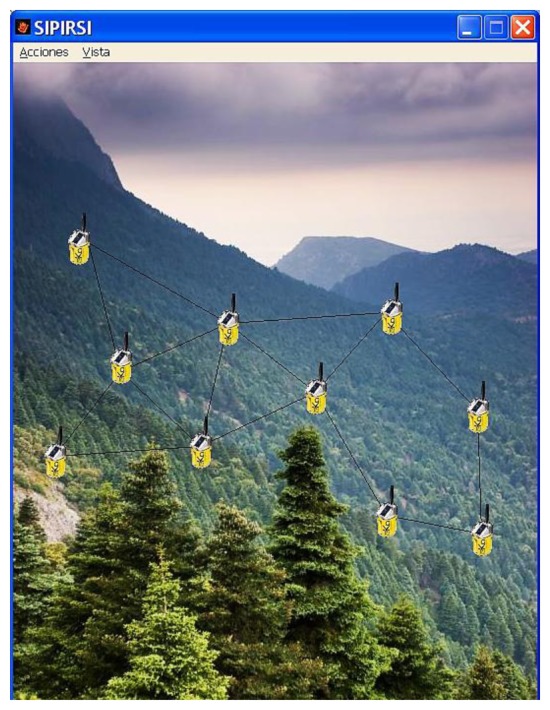
SIPIRSI simulator.

**Figure 13.**
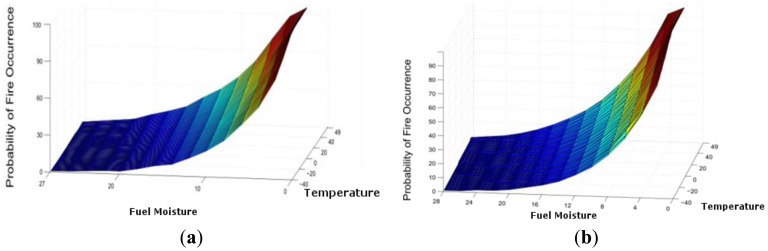
Comparison of the probability of fire. (**a**) SIPIRSI simulator; (**b**) Be-have Plus simulator.

Fortunately, in recent years, no wildfires have occurred in this area, and the accuracy of the system has been tested by using an ad-hoc simulator (Figure 12) that implements the proposed algorithm. This algorithm is compared to the results that were obtained from Be-have Plus, which is a classical tool that is used in forensics fire analysis [20]. In Figure 13, we depict the probability of fire obtained through our system as well as the probability of ignition obtained by the Behave Simulator, respectively. Both figures show the probability as a function of temperature 40–49 °C and fuel moisture 1–27%.

**Figure 14.**
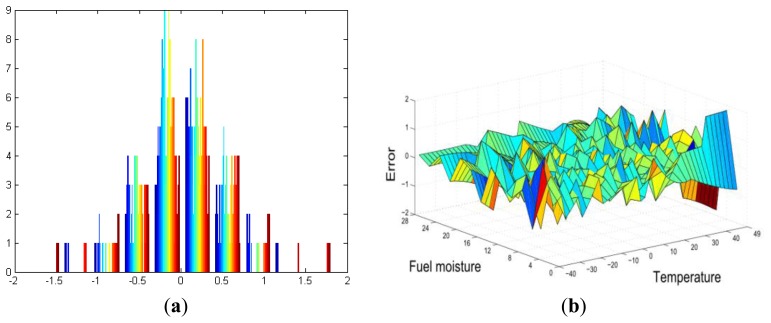
Error comparison. (**a**) Histogram error; (**b**) Dimensional error.

If we compare both results, the average error between them is 0.3588%. Figure 14 depict these errors in the −40–49 °C temperature range.

### 3.4. HORUS Project

**Figure 15.**
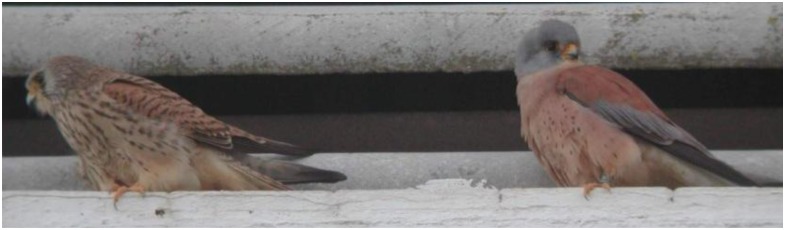
Lesser Kestrel.

In 2011, we started a new collaboration with the DBS on another monitoring sensor network, called HORUS, which is a project that is focused on the observation of behavior in endangered species. Specifically, this project is focused on the phonological monitoring of the Lesser Kestrel (*Falco naumanni*, Figure 15), an endangered small migratory falcon. This species was previously considered to be the most abundant raptor in Europe [21], but then, it became extinct in several countries (e.g., Austria, Hungary, and Poland) and nearly disappeared in others [22] (e.g., France, Portugal, and Bulgaria). Within the western Palearctic, the area with the Mediterranean climate of Spain constitutes a stronghold of the Lesser Kestrel [23]. Nevertheless, the Spanish population has also suffered a precipitous decline in recent years, dropping from an estimated 20,000–50,000 pairs in the 1970s to some 4,000–5,000 breeding pairs in 1988 [24]. This decline has been caused by a reduction in both the extent and the quality of foraging habitats [25].

The observation of animal behavior helps us to understand key issues in ecology, such as which factors influence lifetime reproductive success [26], how parent-offspring conflicts are solved [27,28], which strategies are evolutionarily stable strategies [29] and what are the sex roles in reproduction [30]. This type of study requires a large amount of data that are difficult to obtain using classic methods of animal monitoring because in classic methods, it is necessary to capture individuals repeatedly over short periods of time, which limits the amount and quality of data that can be obtained (captures alter an individual animal's behavior and could jeopardize the survival of the offspring, among other effects).

Traditionally, most of the information about the Lesser Kestrel's behavior has been obtained by direct observation from a distance with telescopes or binoculars, while the body condition of the birds is known via periodic captures of individual birds. However, to obtain conclusions about individual behavior, it is necessary to gather large amounts of data, which normally implies many years of field work because it is not possible to capture animals very often. First, the animals avoid being captured, and most importantly, excessively frequent captures could stress the individuals. This frequent capturing might influence their condition and behavior, reduce the chances of offspring survival if they are breeding.

**Figure 16.**
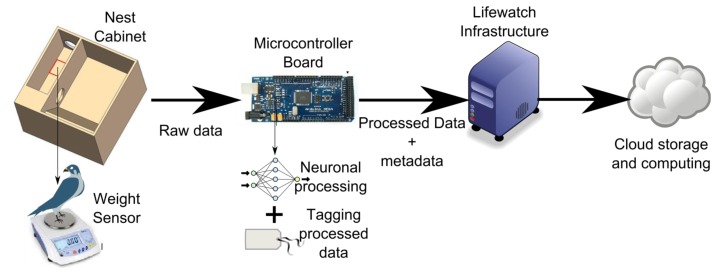
Smart nest architecture.

To overcome these limitations, the HORUS project is based on automatic monitoring [31] using smart-nest boxes (Figure 16) that allow the study of the animals. Each smart-nest box allows us to obtain information, such as the weight of the animal as it enters or leaves the nest. Moreover, the use of RFID tags allows us to distinguish between the different birds in the colony. To estimate the body conditions of the animals, zoologists typically use the body mass corrected by the body size [32]. Other methods that are based on the amount of subcutaneous fat [33], the coloration of feathers or skin [34], or blood chemistry parameters [35] have also been used. However, these methods usually require the capture of individuals, and therefore, they are not suitable for automatic analysis without human intervention. For this reason, the HORUS project is focused on the estimation of the weight of the individuals.

This weight is obtained by using an Artificial Neural Network (ANN) algorithm [36]. This algorithm allows estimating a stable weight value from the acquired unstable weight pattern, which is made by sixteen measurements per second and is obtained while an animal is over the pan of the scale. We used an ANN because the movement of the animals produces unpredictable weight patterns [36]. Some different techniques to estimate the weight have been considered, but the ANN is the technique that produces the most accurate results.

**Figure 17.**
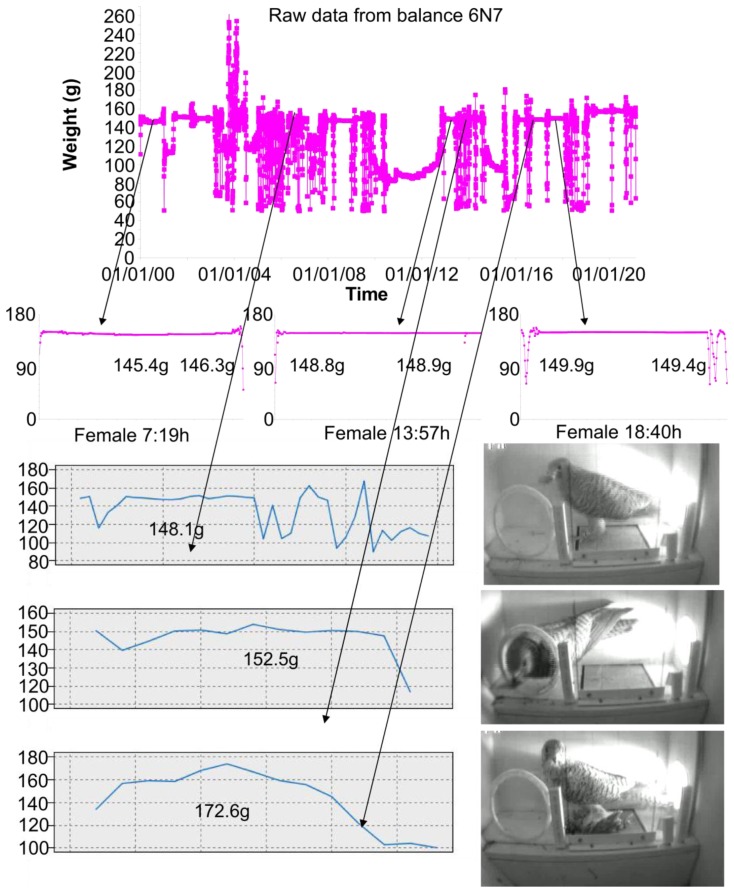
Weight patterns obtained with HORUS.

**Figure 18.**
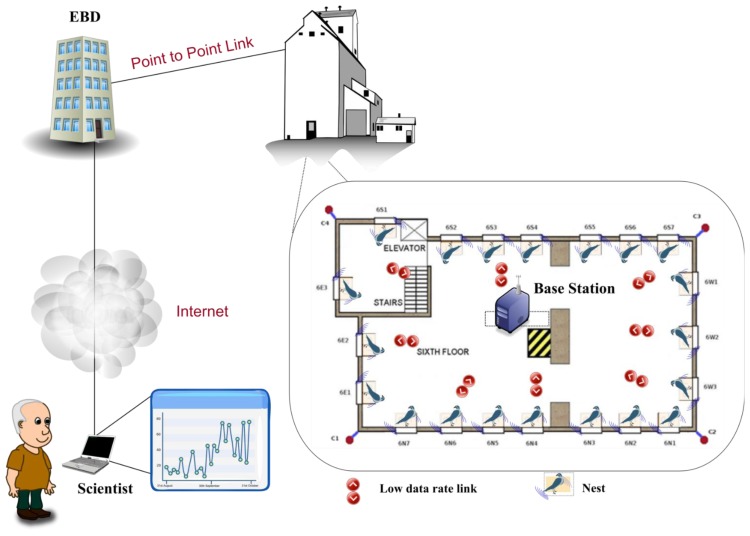
HORUS architecture.

Figure 17 shows some results that are obtained with the system. This system allows us to obtain the weight with an accuracy of 0.1 g. The prototype of this network is implemented in a grain elevator in the surroundings of Doñana; It is composed of a total amount of 20 smart-nest boxes installed in a grain elevator. Figure 18 shows the structure of the network. All of the information that is gathered from the smart-nest boxes is accessible to biologists all over the world through the webpage of the project (http://horus.ebd.csic.es).

### 3.5. ICARO and Networks

ICARO was one of our initial prototypes, and it is described in a previous section. Based on the same architecture, other networks have been designed (*i.e.*, ICARO2, ICARO3, and so on) and cover new areas with sensors to study several environmental variables. These new “ICAROn” networks started to be deployed in 2012, and currently, we are increasing their number, adding extra nodes and sensors. Currently, they integrate the following measurements:

- Temperature semiconductor sensor in the −40–80 °C range.
- Humidity semiconductor sensor in the 0–100% RH range.
- Solar radiation with photodiodes in the 320–730 nm and 320–1,100 nm wavelength ranges.
- CO_2_ using a Non-Dispersive Infrared (NDIR) sensor in the 0–3,000 ppm range.

**Figure 19.**
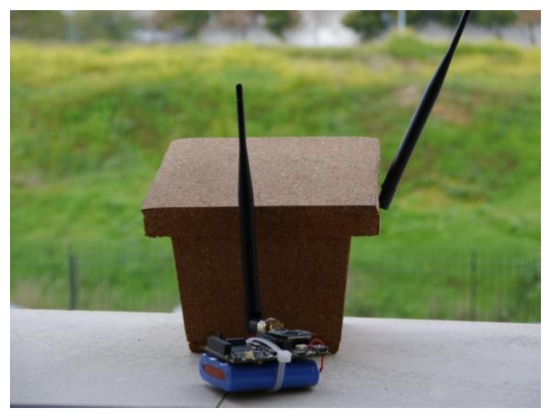
Node of an ICAROn network.

Figure 19 shows a node of an ICAROn network. This node has smaller dimensions than the ICARO 1 node, but it has a similar architecture, which allows the interconnection of a similar number of sensors.

These networks are mainly used to study climate change [37] and its effects on the different areas of the park. The algorithm used to study the climate change is based on a long-term prediction and uses a wavelet transform. We are studying the information that is captured from these sensors, to evaluate the accuracy of their estimations. Depending on the evaluated algorithm, these networks work with a variable sampling rate in 1–1,440 measurements per minute range. Currently, we are working on developing other ICAROn networks in remote locations, to study globally distributed phenomena, such as global change or to monitor migratory birds.

### 3.6. SENDA Network

**Figure 20.**
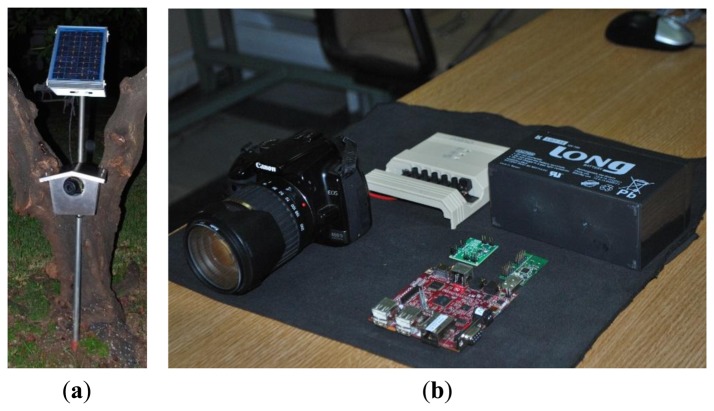
Node of the SENDA network. (**a**) Exterior of the node; (**b**) Devices in the node.

We started to develop the SENDA network in 2012. This network is based on a multimedia sensor network [38], and it is used to monitor animals in flooding areas. Figure 20 depicts a node of these networks and its internal components. The nodes of this network integrate the following sensors:

- A high resolution image sensor (8 megapixel).
- The nodes are designed to support the connection of external high sensitivity microphones.

These devices are based on low-power consumption ARM cortex V7 processors that allow image processing on each node. The nodes collaborate with each other to obtain an enveloping image of a marsh area of Doñana called “Laguna de Santa Olalla”. Because of the very large extent of this area, we must use radio with an extended coverage area (we have, in some cases, more than 500 m between nodes). In this case, we use radio transceivers in the 868 radio range because this range represents a good tradeoff between the radio coverage and the data transmission rate. Decreasing the frequency could improve the coverage, but it drastically reduces the available data bandwidth.

Because of the reduced data bandwidth that is available, only one image is obtained every hour. Nevertheless, the data processing capability of these nodes allows us to monitor the environment because they communicate only the results of the data processing. Currently, we are working on developing efficient image processing algorithms for ARM microprocessors, which will allow us to detect movement and to count animals in the marsh area.

### 3.7. eSAPIENS Network

**Figure 21.**
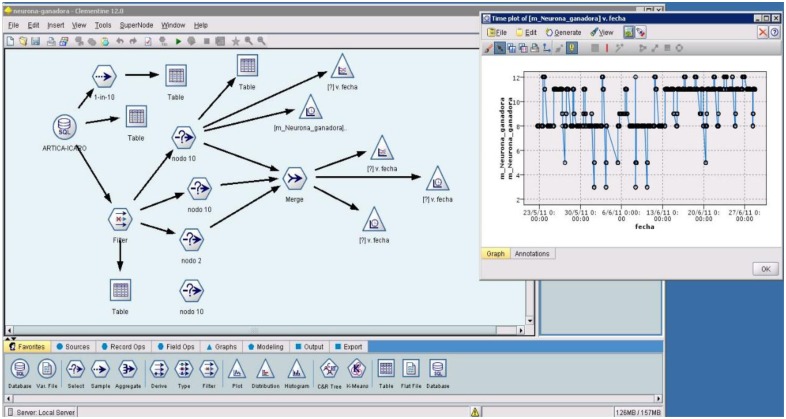
Example of data mining using real-time environmental information on Doñana.

Until 2013, all of the sensor information was gathered from our networks in a classical manner, *i.e.*, each deployed WSN had its own base station that collected sensor node information and sent it to the central server in Doñana, using open-middleware that allowed us to access the information through the internet. The developed middleware allows the representation of the information on a webpage and uses the data with other tools, such as data mining tools (Figure 21).

However, this structure has some scalability problems in very large and widely extended networks. This scheme suffers from several problems: All of the information goes through a Base Station to a single server; the nodes cannot share information directly among the different networks, and therefore, it is not possible to access data directly from its sources (data can be accessed only through the central server).

As an alternative to these classical WSN schemes using a central server to retrieve the information, some authors have proposed a new scheme, which is directly related to the Internet of Things (IoT) philosophy based on the use of IPv6 communications over WSNs [39,40]. Because of the advantages that IPv6 communication schemes add to WSNs, in 2012, we started to migrate all of our networks to this communication scheme.

**Figure 22.**
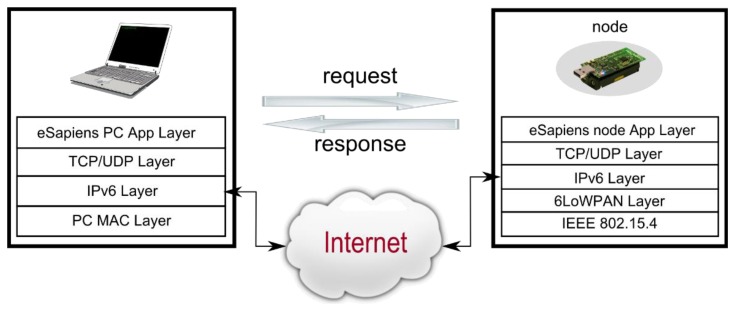
Example of data mining, using real-time environmental information on Doñana.

IPv6 implementations for WSNs (also called 6LoWPAN communications [41,42]) are focused on reducing the requirements of the Base Station as much as possible, *i.e.*, using the base station only as a gateway between the two networks [43], without any intelligence. Because IP is a very common stack, almost any device can be easily used, even standard PCs (Figure 22).

The use of IPv6 provides a common framework in which additional protocols can be added, and a basic interoperability is maintained between networks. The 6LoWPAN implementation not only solves the retrieving information problem from the Base Station but also allows access to every node in the WSN from anywhere on the Internet [44]. Thanks to 6LoWPAN, each node can be uniquely identified [45].

Moreover, IPv6 allows us to add some common communication layers to the network, such as compressed addressing [46] or security layers [47,48]. Using a correct control of the exchange messages, it is possible to increase the efficiency of IPv6 [49,50], which is not initially designed for devices with this reduced data bandwidth.

Due to these 6LowPAN advantages, since 2013, we have been working on interconnecting all of the ICARO networks using this technology, especially focusing on allowing an effective communication infrastructure that permits us to monitor globally distributed problems, such as migratory birds or global climate change.

**Figure 23.**
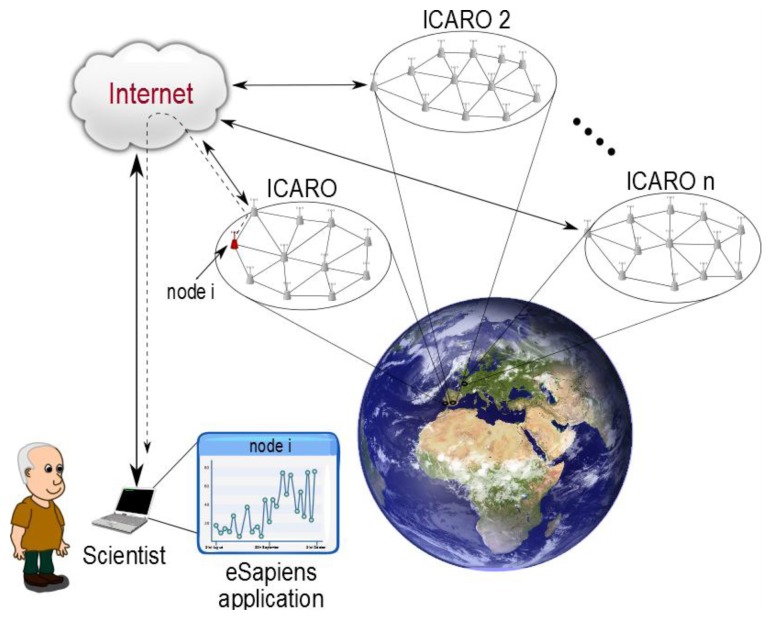
eSAPIENS architecture.

With this infrastructure, we have created a new meta-network called eSapiens, which covers all of the deployed WSNs of Doñana (Figure 23). Moreover, eSapiens allows us to use external WSNs or other services by using the Internet. With eSapiens, each node can communicate directly with other devices, using its individual IP direction.

eSapiens is based on CoAP [51], a UDP protocol that is designed specifically for maintaining the low power consumption of WSNs. With eSapiens, each node stores its own information, allowing direct access to the information through the Internet, *i.e.*, each node acts as a local server, drastically increasing the reliability of the network.

### 3.8. Chronological Evolution of the Prototypes

**Figure 24.**
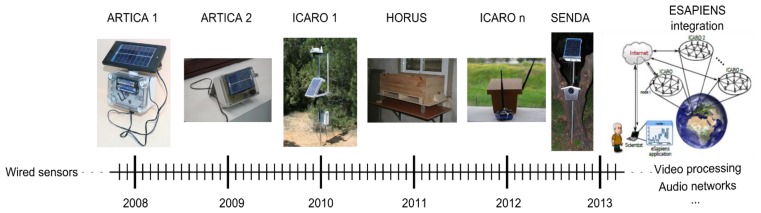
ESAPIENS architecture.

According to the aforementioned information, Figure 24 depicts the chronological evolution of the designed ambient monitoring networks. A comparison of the specification of the different deployed networks is summarized in Table 1.

Currently, all of these networks are working correctly, and they offer valuable information to the biologists working at Doñana. As can be seen, most of the developed networks work at 2.4 GHz, except for SENDA. As described earlier, this arrangement is motivated by the coverage area requirement of this network. According to our practical results, both frequencies can be used for environmental monitoring. The election of a radio frequency requires a tradeoff between reliability and data rate requirements of the application. The 868 MHz radio transceivers have, for environmental monitoring proposes, more coverage area and more reliability than a 2.4 GHz radio transceiver, but with a smaller data rate available.

All of these networks have the name of the project that funds the deployments. Thus far, these projects are the following:

- Project ARTICA: Spanish acronym of “application of sensor networks and computational intelligence techniques for environmental monitoring”.
- Project ICARO: Spanish acronym for “computational intelligence applied to Sensor Networks for environmental observation”.
- Project HORUS: In honor of the Egyptian falcon-god, the Sky god.
- Project SENDA: Acronym of multimedia SEnsor Network for Distributed Animal Observation.
- eSAPIENS: Spanish acronym for “Intelligent acquisition and processing system for natural environments”

## 4. Future WSN Projects in Doñana

In addition to working to improve the networks described above, we are currently devising new networks that are focused on video and audio processing, which are to be installed in the near future. These types of systems are called “multimedia sensor networks” in the literature [34]. These networks are summarized below.

### 4.1. Video Processing of Smart-Nest Boxes

As a continuation of the HORUS project and based on the experience from the SENDA project, we are currently working on designing efficient algorithms to monitor the Lesser Kestrel. Some of the developed smart-nest boxes have integrated video cameras, which are connected by a high-speed local network to the server. These video cameras start recording every time that they detect a movement. However, the main problem is the large amount of information that the system must store. For this reason, an automatic tool is needed that automatically preprocesses and classifies all of the video information.

We are currently designing and developing an automatic monitoring system. The proposed system is based on a combination of a distributed algorithm and a centralized algorithm. The distributed algorithm is based on a low-power consumption ARM board. This board analyzes the video, to detect any useful information. Every time that the system detects any valuable information in the video, it records a 10-second video, compresses it and sends it to the central server. The movement detection in this specific scenario is not a trivial task because the collected frames are difficult to interpret due to a variety of nuisance factors in the data formation process, such as the illumination, vantage point, and partial overlap.

On the other hand, the centralized algorithm is executed in a main central server. This server has more computational resources, and it is responsible for analyzing each video, to obtain some phenological information. Concretely, the algorithm that we have developed can distinguish between the Lesser Kestrel male and females. Moreover, it can detect whether a bird or other species (classified or not) enters the nest. For each individual bird, the automatic video processing analyzes its habits, detecting when it enters or leaves the nest. Moreover, the system can detect if any egg appears in the nest.

The centralized video processing system has been evaluated by using some years of historic information gathered from the HORUS project. According to our preliminary results, the system has a high percentage of success, reducing considerably the biologists' efforts in the manual classification of videos. The proposed system can analyze and classify videos in real time, but classical manual analysis, performed by a biologist, requires several days of work for each day and nest, making the amount of work overwhelming. The system can gather more information that the biologist can process.

### 4.2. Sound Monitoring in Natural Areas

This project constitutes the design of a wireless sensor network that is focused on acquiring and processing audio information. The use of automatic audio pattern recognition in WSNs is not new; some authors have considered it before [52], but it is currently an area under research. In this case, we are focused on the design of an automatic audio processing system that allows us to detect and to distinguish sounds of the different species of frogs that inhabit the marsh areas of Doñana.

The nodes of this network are going to be continuously monitoring the surrounding audio. When the audio increases above a certain audio threshold, the nodes start to process and locally store the pattern. Considering the entire pattern, the system does an initial audio feature extraction to detect if the pattern is a candidate for the species to be recognized. If the pattern matches the desired animal, then the nodes exchange information with each other, detect the best pattern, and send their information, together with the audio level, to the base station. This information is tagged with a time stamp.

Then, the base station processes the received information from the nodes, to localize the audio. This information, together with the time and the pattern, is stored, to allow the biologist to analyze the animal trends.

## 5. Conclusions

In this paper, we present some real WSN prototypes that have been designed to demonstrate the advantages of the use of WSN technology in environmental monitoring in a realistic harsh environment, the Biological Reserve of Doñana, Spain. Currently, these prototypes are fully working and provide valuable information to biologists all over the world.

From the first prototypes that were designed and developed from a laboratory perspective and the recent prototypes that were installed successfully in real scenarios, there is hard work that is yet to be accomplished to reach the final conclusions. Here, we described the most important contributions:

- The strategy used has changed. The first prototypes had, as the main function, to acquire data from the sensors and to send them to a base station. The main disadvantages of this strategy are an intensive use of the radio communication links and, consequently, a high percentage of packet loss and a high level of energy consumption. To avoid these problems, the strategy changed completely to decrease the use of radio communication. Distributed processing schemes have been designed to let the network work as a whole entity that collaborates to accomplish a global goal. In this sense, data aggregation and fusion techniques have been designed and developed.
- The mote designs have been improved. Working in real scenarios implies considering demanding conditions. Problems derived from the effect of radio power absorption by the flora have been considered. The first prototypes operate at the 2.4 GHz band. However, the radio power absorption at this frequency is higher than at lower frequencies. For this reason, the recent prototypes use the 868 MHz band. Another addressed problem is the protection from the effects of weather (rain, wind, humidity, sun, temperature). In this sense, the recent designs have considered better isolation boxes that have a better IP, work according to the IEC 60529 standard, and have a cork coating to prevent extremely high and low temperatures. Furthermore, the electronics components have been selected to work in a wide temperature range.

Currently, the authors are working on increasing the number of nodes that are deployed, designing new software and hardware solutions to monitor specific parameters that are required by biologists, such as the phenological study of vegetation.

## Figures and Tables

**Table 1.** Analysis of the database.

**Project**	**ARTICA 1**	**ARTICA 2**	**ICARO 1**	**Horus**	**ICARO n**	**SENDA**	**eSAPIENS**
Design year	2008	2009	2010	2011	2012	2012	2013
Int. sensors	Temp. Humidity Solar rad.	Temp. Humidity Solar rad.	Tem. Humidity Solar rad. Rainfall Wind dir. Wind speed	Temp. Humidity Weight	Temp. Humidity Solar rad. CO_2_	High resolution image sensor	--
Ext. sensors	Not allowed	Allowed	Not allowed	Not allowed	Allowed	Ext. micro.	Allowed.
Main app.	Monitoring of common environmental variables		Flood level Fire risk Fire tracking Localization	Monitor Lesser Kestrel weight	Climate change Global bird monitoring	Image analysis	Integrate multiple networks
Radio trans.	2.4 GHz IEEE 802.15.4	2.4 GHz IEEE 802.15.4	2.4 GHz IEEE 02.15.4	2.4 GHz IEEE 02.15.4	2.4 GHz IEEE 02.15.4	Ext. range 868 Mhz. radio	Heterogeneous
Main proc.	Low power 16 bit MSP430	Low power 16 bit MSP430	Low power 16 bit MSP430	Low power 16 bit MSP430	Low power 8 bit ATMega	32 bit ARM v7 processor	Low power 16 bit MSP430
Power cons.	85 mW	85 mW	85 mW	85 mW	130 mW	2.5 W	--
Node dist	Up to 200 m	Up to 200 m	Up to 200 m	Up to 200 m	Up to 320 m	Up to 40 km	--
